# A homology guide for Pacific salmon genus *Oncorhynchus* resolves patterns of ohnolog retention, resolution and local adaptation following the salmonid‐specific whole‐genome duplication event

**DOI:** 10.1002/ece3.9994

**Published:** 2023-04-20

**Authors:** Bradford Dimos, Michael Phelps

**Affiliations:** ^1^ Department of Animal Sciences Washington State University Pullman Washington USA

**Keywords:** ohnolog, salmonid, SS4R, whole genome duplication

## Abstract

Salmonid fishes have emerged as a tractable model to study whole‐genome duplications (WGDs) as this group has undergone four rounds of WGDs. While most of the salmonid genome has returned to a diploid state, a significant proportion of genes are maintained as duplicates and are referred to as ohnologs. The fact that much of the modern salmonid gene repertoire is comprised of ohnologs, while other genes have returned to their singleton state creates complications for genetic studies by obscuring homology relationships. The difficulty this creates is particularly prominent in Pacific salmonids belonging to genus *Oncorhynchus* who are the focus of intense genetics‐based conservation and management efforts owing to the important ecological and cultural roles these fish play. To address this gap, we generated a homology guide for six species of *Oncorhynchus* with available genomes and used this guide to describe patterns of ohnolog retention and resolution. Overall, we find that ohnologs comprise approximately half of each species modern gene repertoires, which are functionally enriched for genes involved in DNA binding, while the less numerous singleton genes are heavily enriched in dosage‐sensitive processes such as mitochondrial metabolism. Additionally, by reanalyzing published expression data from locally adapted strains of *O. mykiss*, we show that numerous ohnologs exhibit adaptive expression profiles; however, ohnologs are not more likely to display adaptive signatures than either paralogs or singletons. Finally, we demonstrate the utility of our homology guide by investigating the evolutionary relationship among genes highlighted as playing a role in salmonid life‐history traits or gene editing targets.

## Graphical abstract

We described how the salmonid‐specific whole‐genome duplication has influenced the gene repertoires of six species of *Oncorhynchus*. Overall, we found that gene function is a major driver of whether a gene will be retained as an ohnolog or it will revert to a singleton state, which in turn influences chromosomal synteny. We also produced a multi‐species homology guide which we hope will aid in the field of salmonid genetics.

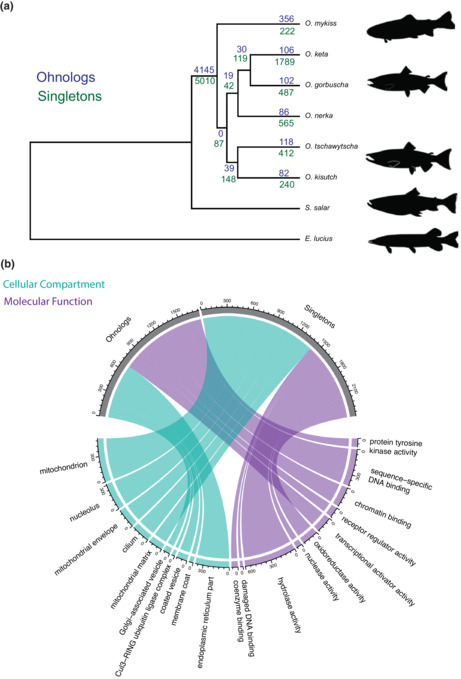

## 1 INTRODUCTION

Whole‐genome duplications (WGDs) have taken place many times during eukaryotic evolution (Otto & Whitton, 2000), which includes vertebrates, whose common ancestor underwent two rounds of whole genome duplication referred to as the 2R hypothesis (Dehal & Boore, 2005). Some vertebrate taxa have undergone additional rounds of WGD, including a third round in teleost fish (Jaillon et al., 2004) and a fourth round in the common ancestor of salmonids termed the SS4R (Lien et al., 2016). Initially, WGD events were studied in plants and thought to promote genomic instability and rearrangement (Pontes et al., 2004) leading to an evolutionary dead‐end (Mayrose et al., 2011). However, work in amphioxus does not support WGDs as a source of instability (Hufton et al., 2008), and evidence suggests that WGDs may be a major driver of evolution and diversification (Soltis et al., 2014). In fact, WGDs can promote adaptation through the simultaneous duplication of all genes, thereby creating large‐scale opportunities for gene neofunctionalization and subfunctionalization (Force et al., 1999; Lynch, 2000; Ohno, 1970). However, whether neofunctionalization or subfunctionalization predominate following a WGD has proved difficult to quantitatively address (Sandve et al., 2018) as has the role WGDs play in promoting adaptation. Salmonid fishes have emerged as a tractable model to study WGDs as a substantial proportion of their genome has retained high sequence similarity between duplicate regions despite returning to a diploid state in the 80‐106MYA since the SS4R (Gundappa et al., 2022; Lien et al., 2016).

Salmonid fishes including those in the genera *Salmo*, *Oncorhynchus*, *Hucho*, *Thymallus*, and *Salvelinus* have provided insights into evolution following the SS4R as genome assemblies exist for many of these species (Christensen et al., 2018; Gao et al., 2021; Gundappa et al., 2022; Lien et al., 2016; Sävilammi et al., 2019). Rediploidization, the process of moving from tetravalent pairing during meiosis toward bivalent pairing, is ongoing in salmonids and proceeds in bursts. Under this model, an initial wave of rediploidization occurred in the common ancestor of modern salmonids immediately following the WGD, followed by a period of relative stasis, and then, a smaller wave of lineage‐specific rediploidization occurred (Gundappa et al., 2022). The process of rediploidization is driven by structural rearrangements such as transposable element insertions, which reduce homology and inhibit chromosomal pairing (Lien et al., 2016). The modern salmonid genome has largely rediploidized, as only a limited proportion of the genome engages in tetravalent pairing (Campbell et al., 2019; Lien et al., 2016). Importantly, rediploidization allows for ohnologs (gene duplicates produced from a WGD event) to acquire new functions as recombination between the gene copies is disrupted allowing for sequence and potentially functional divergence between the copies. Ohnologs differ from paralogs as the former are the result of retention of gene duplicates produced from a WGD, while paralogs are retained duplicates arising from gene‐level duplication. In salmonids, many ohnologs have developed divergent expression patterns and thus are thought to be protected from gene loss and a reversion to a singleton state via pseudofunctionalization due to functional constraints (Gillard et al., 2021). However, whether this regulatory divergence occurs due to neofunctionalization or relaxed purifying selection remains an open question (Sandve et al., 2018).

In addition to their importance in understanding genome evolution, salmonid fishes are also of tremendous cultural, economic, and ecological significance. In North America, Pacific salmonid populations in the genus *Oncorhynchus* have provided food for indigenous communities since time immemorial (Atlas et al., 2021) and are a critical link for the flow of nutrients between the ocean and terrestrial ecosystems (Schindler et al., 2003). However, many populations of *Oncorhynchus* are experiencing precipitous declines (Crozier et al., 2007; Waldman & Quinn, 2022). These declines have prompted intense interest in understanding the genetic basis of ecologically important traits and the development of genome editing technology to aid in conservation efforts (Phelps et al., 2020; Waples et al., 2020). However, the variation of gene copy number produced by differential ohnolog retention and resolution to the singleton state between species and inconsistent gene naming complicates these efforts (Limborg et al., 2017; Rougemont et al., 2022) by making the identification of homologous genes, a prerequisite for either genetic analysis or genome editing efforts, extremely challenging.

Given the substantial influence copy number variation can have on ecologically important traits and evolutionary patterns, we investigated ohnolog retention and resolution to a singleton state across six Pacific salmon species of the genus *Oncorhynchus* with sequenced genomes: Chinook (*O. tshawytscha*), Coho (*O. kisutch*), Sockeye (*O. nerka*), Chum (*O. keta*), Pink (*O. gorbuscha*), and Rainbow trout (*O. mykiss*). For three species Chinook, Sockeye, and Rainbow trout, we investigated how ohnolog retention/resolution has influenced genome structure. Additionally, we examined the potential of ohnologs to facilitate adaptation by reanalyzing gene expression from the redband Rainbow trout model system (*O. mykiss gairdneri*) (Chen et al., 2018). Through our investigation, we produced a gene family‐based homology guide for *Oncorhynchus* which describes the ohnolog/paralog/singleton relationship for each gene in the genome together with both gene name (NCBI ID), copy number, and a consensus annotation that we hope we will serve as an easy to use resource for the salmonid community to address the issue of homolog identification. To demonstrate the utility of our homology guide, we produce gene trees for homologous gene families highlighted as influencing life‐history variation or potential high‐value gene editing targets.

## 2 RESULTS AND DISCUSSION

### 2.1 Gene family evolution following the SS4R

In order to understand the process of ohnolog retention and resolution in *Oncorhynchus*, we grouped genes into homologous genes families and quantified the copy number for each of the six species with publicly available genomes at the time of analysis: *O. tshawytscha*, *O. kisutch*, *O. mykiss*, *O. nerka*, *O. keta*, *O. gorbuscha*, as well as Atlantic salmon (*Salmo salar*) and Northern Pike (*Esox lucius*) as outgroups. Gene families were assigned into one of the six categories: ohnologs, paralogs, singletons, resolving, multi‐copy expanding, or contracting based on comparison to *E. lucius*. As pointed out by the reviewer, the multi‐copy expanding gene families represent a subcategory of ohnologs, which have undergone additional duplications and thus contain both ohnologs and paralogs, which we are not able to differentiate via our methodology. The contracting gene families meanwhile represent a subcategory of singletons which either have undergone additional gene losses or could have duplicated in the outgroup (*E. lucius*). To differentiate between ohnologs and paralogs (which would have the same copy number), we determined whether the duplicate copies were on homoeologous chromosomes previously determined for each species (Christensen et al., 2018, 2020, 2021; Pearse et al., 2019; Rondeau et al., 2021, 2022). If the duplicates were on homoeologous chromosomes, they were called as ohnologs, while if not or if one of the gene copies was not assigned to a chromosome, it was called a paralog. Additionally, our classification of resolved to a singleton state indicates that the gene family copy number has resumed the pre‐SS4R copy number found in *E. lucius*. The genome of *E. lucius* encompasses both single‐copy and multi‐copy gene families derived from the teleost‐specific WGD.

**FIGURE 1:**
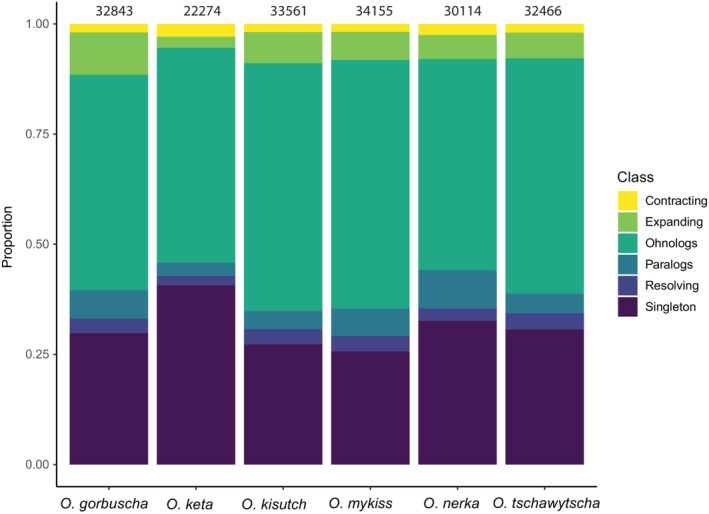
Relative proportion of the gene repertoire of each species belonging to each of the six categories with gene number of species listed above bar.

The number of gene families which are either ohnologs, singletons, resolving, expanding, or contracting varied some by species but were relatively consistent with the exception of *O. keta* (Figure 1). The difference seen in *O. keta* were likely due to reduced completeness and lower contiguity of this genome assembly as noted by the authors (Rondeau et al., 2021). Ohnologs were the most common class of genes representing between 48% and 56% of all genes, followed by singletons which varied between 26% and 40% of all genes (Figure 1). Interestingly, we found that only around 2.13%–3.65% of homologous genes were in the process of resolving. Expanding gene families made up between 2.50% and 9.47% of all homologous genes, while contracting gene families comprised between 1.92% and 2.90% of all homologous genes. Overall, these results demonstrate the magnitude of the influence the SS4R played in shaping the current gene repertoires of *Oncorhynchus* spp. For a full breakdown of the percentage of gene families within each category, see Table 1.

**TABLE 1:** Listed are the number of genes in each gene category broken down by species.

Species	Contracting	Expanding	Singleton	Resolving	Ohnologs	Paralogs
*Oncorhynchus gorbuscha*	652	3220	10,012	1092	16,382	2158
*Oncorhynchus keta*	658	566	9208	482	11,040	658
*Oncorhynchus kisutch*	638	2421	9339	1203	19,252	1388
*Oncorhynchus mykiss*	633	2238	8990	1222	19,696	2168
*Oncorhynchus nerka*	764	1679	9969	853	14,630	2636
*Oncorhynchus tshawytscha*	661	1927	10,157	1215	17,670	1478

### 2.2 Ohnologs

**FIGURE 2:**
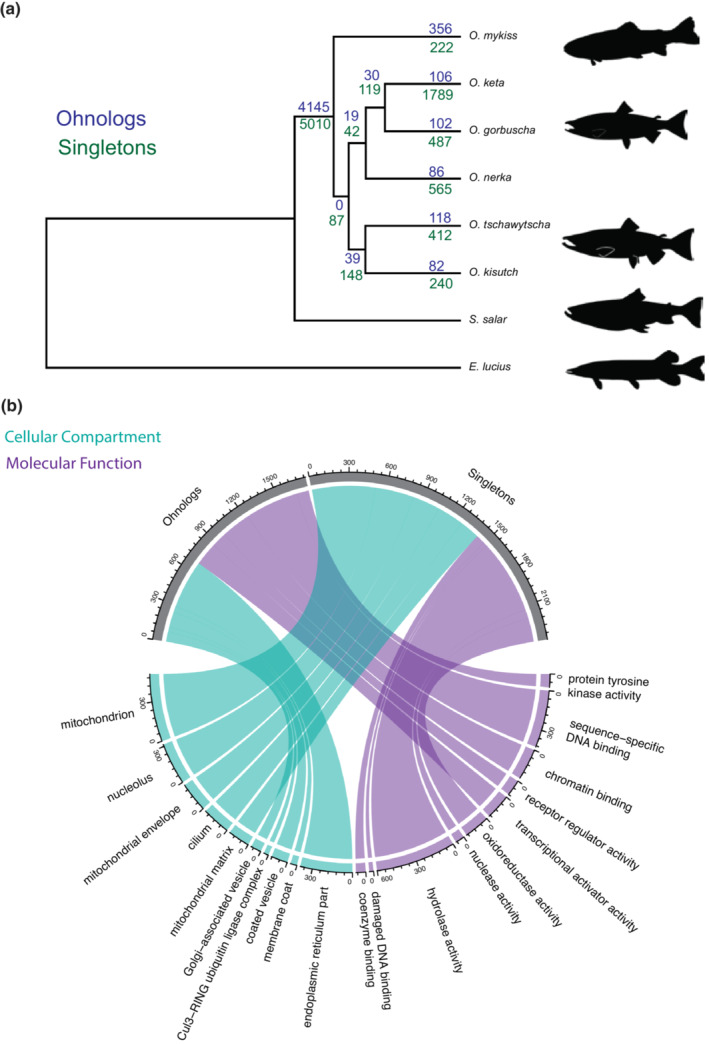
Patterns of ohnolog retention and resolution to a singleton state. (a) Species Tree with the number of genes families which are retained ohnologs (blue) or have reverted to a singleton state (green). (b) Chord diagram showing the visualization of select GO terms, where GO terms are linked to either universally retained ohnologs or universally singleton gene families. Size of chord is proportional to the number of genes in a term which are either ohnologs or singletons. Color denotes cellular compartment versus molecular function. For a full list of enrichments, see Table S1. Silhouettes were obtained from (http://phylopic.org).

The high number of ohnologs is notable given that most gene duplicates are expected to be purged quickly following WGD (Innan & Kondrashov, 2010). However, if gene duplicates acquire new functions through sequence evolution or regulatory divergence (Force et al., 1999; Kondrashov, 2012), they can persist for millions of years. Interestingly, we observe high overlap in the number of ohnologs shared between species. Of the 11,279 ohnologs present in at least one species, 4145 are retained universally across the six *Oncorhynchus* species. Species‐specific ohnologs were less common, ranging from 82 in *O. kisutch* to 356 gene families in *O. mykiss* (Figure 2a).

Given that almost 40% of ohnologs are retained universally, this suggests that constraints on gene function (Harper et al., 2021) could be maintaining these genes as ohnologs in *Oncorhynchus*. To test this theory, we performed Gene Ontology (GO) enrichments on the universally retained ohnologs. Our analysis revealed 27 cellular compartment enrichments, which are involved in various functions such as “endoplasmic reticulum part,” “subsynaptic reticulum,” and “polytene chromosome puff” (Figure 2b, Table S1). Likewise, the universally retained ohnologs were also enriched for 37 molecular function terms focused on DNA binding including, “chromatin binding,” “sequence‐specific DNA binding,” and “transcriptional activator activity.” However, there are also several other processes enriched among this list, including “ephrin receptor binding,” “chemorepellent activity,” and “sphingolipid binding” (Figure 2b, Table S1). Given the essential role of these processes in basic cellular integrity and genomic stability, our results raise the possibility that these genes may be maintained as ohnologs due to their core functional significance. To this point, the observed uniform copy number in all six salmonid species is unlikely to have been maintained across the 106 MYA (Gundappa et al., 2022) since the SS4R, unless the loss of one gene copy is highly selected against, supporting gene function as a potential driver of why a gene family may be maintained as ohnologs.

### 2.3 Singleton genes

Together, ohnologs and singleton genes make up over 79% of all genes in the salmonid genome (Figure 1). Interestingly, we identified a limited number of multi‐copy genes families that were an intermediate size between the outgroup and what would be expected if all ohnologs were maintained meaning that loss of ohnologs had occurred; however, the gene families had not attained the pre‐SS4R copy number. This intriguing finding suggests that either the process of ohnolog resolution back to a singleton state in *Oncorhynchus* may be nearly complete, or alternatively that this process can only be tolerated at a certain number of gene families simultaneously.

In salmonids, rediploidization occurs in bursts, where the first rediploidization event occurred in the common ancestor of salmonids, followed by a period of relative stasis, where another more recent round of rediploidization occurred in tandem with species diversification (Gundappa et al., 2022). Ohnologs can only return to a singleton state after the genomic region on which they are located has rediploidized as the elevated recombination rate of tetraploid regions should efficiently remove mutations which would cause a gene to become nonfunctional and return to a singleton state (McGrath et al., 2014). Thus, patterns of singleton gene occurrence should follow patterns of rediploidization. Our results support this, as of the 14,776 total gene families which are singletons in at least one species, 5010 are universally singletons in all six species (Figure 2a), while species‐specific singletons are less common, ranging from 222 in *O mykiss* to 1789 in *O. keta* (Figure 2a). While the number of gene families which have resolved to singletons is larger than the number which are retained as ohnologs the copy number of the singleton gene families is lower and thus affects a smaller number of genes.

To determine what processes are affected by ohnolog resolution to a singleton state we performed GO enrichments on the universally singleton gene families. These enrichment analyses revealed 28 enriched cellular compartment terms, which relate to mitochondria and DNA including, “mitochondrion,” “mitochondrial matrix,” “DNA repair complex,” and “replication fork” (Figure 2b, Table S1). The universally singleton genes were also enriched for 26 molecular function terms including “damaged DNA binding,” “oxidoreductase activity,” “tRNA binding,” and “ligase activity” (Figure 2b, Table S1). The return to a singleton state of genes following a WGD is hypothesized to be driven by dosage balance constraints (Makino & McLysaght, 2010) and thus dosage‐sensitive genes would be expected to attain the gene copy number of the singleton (Woodhouse et al., 2010). Taken together, our data support the link between dosage sensitivity and ohnolog resolution to a singleton state as the largest categories of enrichments are highly dosage‐sensitive processes such as mitochondrial metabolism (Toivonen et al., 2003). Mitochondria are particularly sensitive to gene dosage as mitochondrial activity requires the careful coordination of both the mitochondrial and nuclear genome. Ensuring appropriate stoichiometric balance between the mitochondrial and nuclear genomes is critical for mitochondrial function (Wang et al., 2020). In fact, there are dedicated cellular pathways to ensure that both genomes work in harmony, which are conserved across animal evolution (Dimos et al., 2019). WGDs thus have the potential to cause imbalance between nuclear and cytoplasmic genomes. Some polyploid plants solve the problem of stoichiometric balance through increasing the organellar genome copy number (Fernandes Gyorfy et al., 2021). However, our data indicate that the common ancestor of *Oncorhynchus* resolved mitochondrial ohnologs in a manner consistent to achieve dosage balance between the nuclear and mitochondrial genome copy numbers. Interestingly, it has been shown that *S. salar* downregulates the expression of one copy of mitochondrial ohnologs (Gillard et al., 2021); thus, it appears that salmonids resolved a large number of nuclear‐encoded mitochondrial genes shortly after the SS4R and those that did not return to a singleton state developed compensatory expression patterns to avoid stoichiometric imbalance.

In addition to nuclear‐encoded mitochondrial genes, several other notable categories of genes, mostly related to nucleic acid repair, are universally singleton across *Oncorhynchus* including those involved in DNA repair, replication fork, co‐enzyme binding, tRNA binding, and nuclease activity. While speculative, these processes might also be dosage‐sensitive and thus favor resolution to a singleton state, since they often involve the coordinated activity of many proteins. For example, tRNA copy number plays a role in determining codon usage (Duret, 2000) and processes such as DNA repair are sensitive to gene dosage (Chae et al., 2016). Overall, given the patterns observed both among the universally retained ohnologs and the universally singleton gene families, functional constraints on genes appear to be a major determinant of whether a gene family will be retained in its duplicate state following a WGD.

### 2.4 The influence of ohnolog retention and resolution on genome structure

As rediploidization following a WGD is predicted to promote genomic instability (Semon & Wolfe, 2006) through structural rearrangements or transposable element insertion (Lien et al., 2016), we investigated whether such a relationship can be observed in Pacific salmon at the gene family level. To address this question, we identified syntenic (blocks of chromosomal homology between species) regions between *O. mykiss*, *O. tshawytscha*, *O. nerka*, and *S. salar* and tested whether ohnologs or singleton gene families were more or less likely to occur in syntenic regions. As genomic rediploidization is driven by structural rearrangements such as transposable element insertions (Lien et al., 2016; Sémon & Wolfe, 2007), we would expect ohnologs to be more likely to occur in more stable syntenic regions and singletons to be more likely occur in less stable regions where syntenic relationships have been disrupted.

**FIGURE 3:**
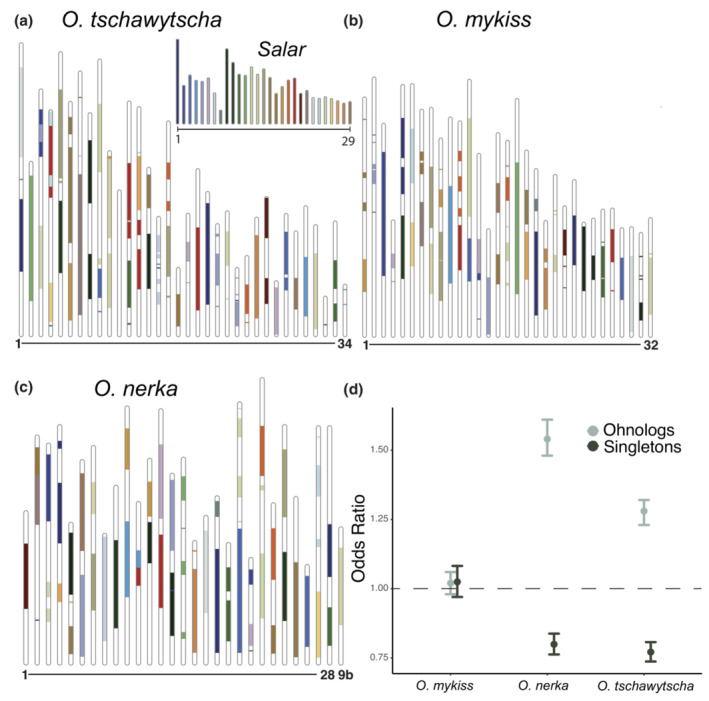
Synteny map for (a) *O. tshawytscha*, (b) *O. mykiss* and (c) *O. nerka* compared with *S. salar* (inset). (d) Odds ratio with 95% confidence intervals for the relationship between ohnologs, singleton genes, and syntenic regions for each species.

Overall, we observe high levels of genomic synteny as 38.9% of the *O. mykiss* genome, 49.2% of the *O. tshawytscha* genome and 48.2% of the *O. nerka* genome have obvious syntenic relationships with the *S. salar* genome (Figure 3a–c). Additionally, syntenic regions are found on every chromosome/linkage group with the exception of *O. tshawytscha* linkage group 11. While it is not apparent why *O. mykiss* has reduced levels of synteny compared with the other two species, two potential factors are that the *O. mykiss* genome used here (Arlee) was based on a clonal individual (Gao et al., 2021), which could lead to synteny‐disrupting structural variants that would not be expected to occur in wild populations or that the karyotype of *O. mykiss* is not fixed (Thorgaard, 1983). Ohnologs were more likely to occur in syntenic regions than a size‐matched random downsampling of genes in both *O. tshawytscha* (odds ratio 1.28, *p*‐value <2.2e−16; Fisher's exact test) and *O. nerka* (odds ratio 1.540, *p*‐value <2.2e−16; Fisher's exact test), but not for *O. mykiss* (odds ratio 1.02, *p*‐value .277; Fisher's exact test) (Figure 3d). Likewise, singleton genes were less likely to occur in syntenic regions (Figure 3d) in *O. tshawytscha* (odds ratio 0.921, *p*‐value = .00212; Fisher's exact test) and *O. nerka* (odds ratio 0.921, *p*‐value = .00328; Fisher's exact test), but not *O. mykiss* (odds ratio 1.02, *p*‐value = .383; Fisher's exact test) compared with a random downsampling of genes.

We find general support for the relationship between genome synteny and ohnolog retention and resolution. Taken together with the observed gene function enrichment patterns, our data provide support for gene function as a likely determinant of whether a gene family will be retained as an ohnolog, or will be resolved into a singleton state, which in turn may influence genomic structure. It should be noted, however, that our analysis is unable to determine whether ohnolog retention/resolution causes genomic rearrangements or visa‐versa. To this point, if chromosomal rearrangements or transposable element insertions lead to loss of one of the ohnolog copies, then genomic rearrangement would be the driver of rediploidization. Supporting a role for gene function, genes which are involved in DNA stability seem to be particularly likely to be retained as ohnologs, while genes involved in mitochondrial metabolism are more likely to have attained the copy number of the singletons. Interestingly, both ohnologs and singleton genes which are shared between the six species of *Oncorhynchus* are far more numerous than those which are species‐specific. The universally retained ohnologs appear to likely be highly refractory to copy number change as the copy number of these families have been maintained since the SS4R. For the universally singleton gene families, the increased dosage following the SS4R was likely highly deleterious and organismal viability following the WGD was likely dependent on rapid resolution of ohnologs to restore the copy number of the gene families.

### 2.5 Ohnologs have a limited association with adaptive divergence

**FIGURE 4:**
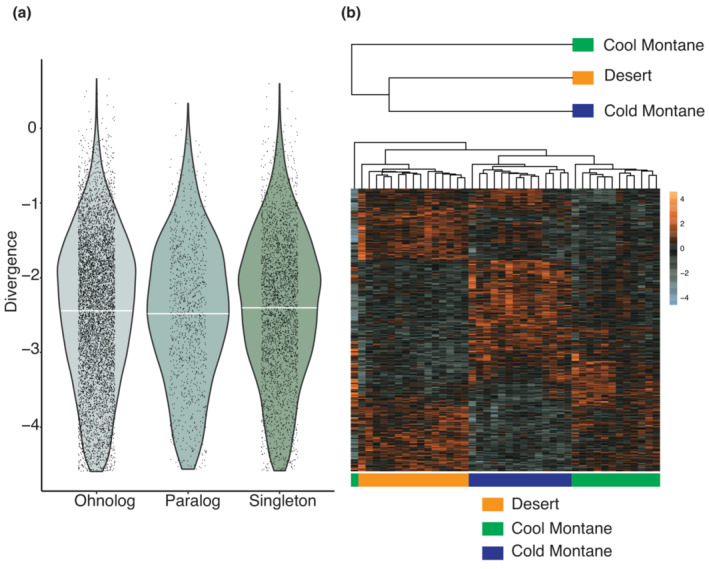
Locally adaptive expression. (a) Violin plots showing the divergence level of each gene belonging to the categories of ohnologs, paralogs, or singletons. White line shows the mean of each category. The divergence metric is derived from a ratio and thus is a unitless metric, where genes closer to zero have higher levels of expression divergence and genes with more negative values have lower levels of expression divergence. (b) Heatmap shows the *z*‐score normalized expression of the 379 ohnologs which show divergent expression profiles between cold montane, cool montane, and warm desert streams. Each column is a sample where population is denoted by color and each row is a gene. Orange gradient is increased level of gene expression whereas gray is decreased level of gene expression. Inset shows the phylogeny used in the EVE model.

Ohnologs have been widely suggested to promote adaptation; however, only a few empirical examples exist which support this relationship in animals. To investigate this process, we analyzed RNA‐Seq data taken from heart ventricles from three recently diverged populations of Redband trout (*O. mykiss gairdneri*), which have adapted to live in cold montane, cool montane, or warm desert streams (Narum et al., 2010; Narum & Campbell, 2015). To investigate the relationship between ohnologs and adaptation, we used the Expression Variance and Evolution model (EVE) (Gillard et al., 2021) to identify genes which have divergent expression patterns between populations. Of the 25,140 genes used in the model, 783 genes displayed divergent expression patterns (*p* < .05), of which 379 are ohnologs, 52 are paralogs and 169 are singletons, while the remaining genes fall under the categories of multi‐copy expanding or contracting. However, no significant differences exist in expression divergence between these three classes (*p* > .1; one‐way ANOVA with Tukey's HSD) (Figure 4a). While the original implementation of the EVE model did not employ multiple testing correction, at the suggestion of the reviewers, we applied a false discovery rate correction where a gene was considered significant at an FDR < 0.1. This resulted in a total of 42 genes which display divergent expression patterns which included five ohnologs, one paralog, and 10 singletons, which likewise displayed no differences between groups (*p* > .1; one‐way ANOVA with Tukey's HSD). We found no evidence that our approach had difficultly differentiating between ohnolog gene copies due to low sequence divergence as the proportion of ohnologs expressed above the minimum threshold was similar to the proportion of singleton genes.

Interestingly, the proportion of ohnologs, which display adaptive expression patterns is similar to the proportion of these gene classes genome‐wide (odd ratio = 0.957, *p*‐value = .556), suggesting that the emergence of adaptive expression in this tissue is stochastic and not dependent on status as an ohnolog. These results are surprising as under the classic model of gene duplication, additional copies should only be maintained if they provide some selective benefit such as neofunctionalization, subfunctionalization, or adaptive changes in gene dose (Force et al., 1999; Ohno, 1970); however, if ohnologs were selectively neutral, they could still be maintained in the absence of adaptation. While it is not surprising that ohnologs are not overrepresented as they comprise nearly half of the modern *O. mykiss* gene repertoire, the similar proportions of singletons and paralogs demonstrating divergent expression is unexpected. It is important to note that our results are based on the gene expression profiles of a single tissue (ventricle) (Chen et al., 2018), and thus, we are only observing a portion of locally adapted genes and it is possible that some of the paralogs/ohnologs have adaptive functions in other tissues not sampled here. Furthermore, we are only looking at expression levels, which would not identify adaptation at the protein‐coding level.

While our results demonstrate that ohnologs are not more likely than other gene classes such as singletons or paralogs to develop divergent expression patterns between populations, the ohnologs with divergent expression patterns show clear differences in expression between the desert and cold montane populations, with cool montane populations showing an intermediate expression profile (Figure 4b). Thus, while as a class ohnologs are not more likely to demonstrate expression divergence, the 379 ohnologs which do exhibit this pattern provide support for the theory that ohnologs can provide the raw genetic material to promote adaptive expression patterns. The recognition of WGDs as drivers of evolutionary adaptation is increasing (Glasauer & Neuhauss, 2014; Moriyama & Koshiba‐Takeuchi, 2018) with the demonstration that some evolutionary novelties such as the development of electric organs in some fishes and the development of the bulbus arteriosus in zebrafish are due to functional divergence between ohnologs (Moriyama et al., 2016; Zakon et al., 2006). Recently, divergent expression of ohnolog copies was likewise demonstrated to promote adaptation to salt water in mangrove plants (Xu et al., 2021). Overall, while our analysis of published data demonstrates the potential ohnologs to develop adaptive expression patterns, this is likely the result of an increased number of genes rather than a special propensity of ohnologs to functional diverge.

### 2.6 Homology guide utility

While salmonids are an ideal model to study evolution following a WGD, these fish are also intensely studied from a conservation and management perspective. Genetics is an essential component to modern salmonid management; however, variation in ploidy generated from the SS4R is difficult to incorporate (Limborg et al., 2017; Narum et al., 2018), due to the paucity of data on gene copy number coupled with inconsistent naming schemes. Thus, we hope that our homology guide differentiating between ohnolog/paralog/singleton genes together with corresponding NCBI gene IDs and common annotations across these six species of *Oncorhynchus* will prove to be a valuable resource for the Pacific salmonid research community. As an example, we demonstrate how this homology guide can be used to aid in the context of both genome‐wide association studies and the emerging field of genome editing by illustrating how the SS4R has influenced the copy number and evolutionary relationships determined by Orthofinder2 of four previously highlighted gene families: *greb1l*, *vgll3*, *slc45a2*, and *dnd1*.

**FIGURE 5:**
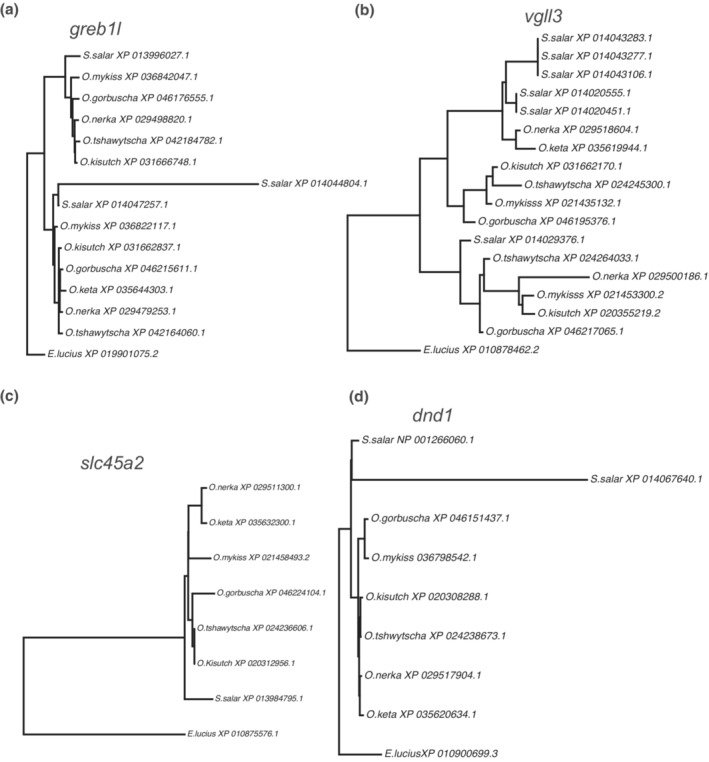
Gene trees of the genes highlighted as either being involved in life‐history variation; (a) *grebl1* and (b) *vgll3*, or as targets for gene editing; (c) *slc45a2* and (d) *dnd1*.

Run timing of coastal *O. mykiss* and *O. tshawytscha* in the western United States was shown to be nearly perfectly associated with variation in a genomic region containing the *greb1l* gene on chr. 28 (Hess et al., 2016; Prince et al., 2017; Thompson et al., 2020), and this gene has also been associated with age at maturity in *S. salar* (Cauwelier et al., 2018). The gene *greb1l* is represented by gene family 5379 and is double copy across *Oncorhynchus*, except for being single copy in *O. keta*, and triple copy in *S. salar* (Figure 5a). The *greb1l* gene tree represents a standard ohnolog (except for the gene loss in *O. keta* and the extra duplication in *S. salar*), as each version of the ohnolog forms a monophyletic cluster. While the *greb1l* copy associated with run timing is located on chr. 28, there is another *greb1l* copy located on chr. 10. This raises the question of the functional significance of the other *greb1l* copy and whether the ability of the *greb1l* copy on chr. 28 to determine run timing may be an example of neofunctionalization.

Age at maturity is an ecologically important life‐history trait and has recently been shown to be associated with *vgll3* in *S. salar* (Ayllon et al., 2015). The gene *vgll3* is represented by gene family 1099 and has the same copy number as *greb1l* with the exception of having six copies in *S. salar* (Figure 5b) and has an unclear evolutionary relationship likely due to the proliferation of the gene family in *S. salar*. Interestingly, the function of *vgll3* is not conserved between the two genera as *vgll3* has no influence on age at maturity in *Oncorhynchus* (Waters et al., 2021) and thus the ability of *vgll3* to determine age at maturity may be an example of neofunctionalization of one of the duplicates in *S. salar*. This example underlies how gene copy number can influence function as well as the importance of incorporating ploidy in genome‐based association studies for organisms which have experienced WGDs. As the field of ecological genomics continues to unravel more aspects of salmonid biology, we hope that our homology guide will prove to be a useful resource to translate findings across species.

A method gaining traction for functional genomics research or as a conservation tool is gene editing through the use of CRISPR technologies (Phelps et al., 2020). For CRISPR technologies, it is particularly important to understand gene copy number and homology. Without this information, an edited animal may show no phenotypic differences due to the activity of a redundant gene copy, or alternatively a CRISPR targeting guide RNA may have multiple off‐target effects if copy number is not accounted for. Salmonids have proven to be amenable to the use of CRISPR technology and the pigment transporter gene *slc45a2* is commonly used as a marker gene to screen for an albino phenotype in edited fish (Edvardsen et al., 2014; Straume et al., 2020). As an example of the utility of the homology guide, *slc45a2* represented by gene family 16,929 is single copy in all species (Figure 5c) and the gene tree of *slc45a2* is characteristic of a universally singleton gene. Thus, this gene will likely serve as an effective marker gene for editing in any species of *Oncorhynchus*. Another example is the dead‐end gene (*dnd1*), which has been edited in *S. salar* and *O. mykiss* with the intent to produce fish with inherited sterility (Fujihara et al., 2022; Güralp et al., 2020; Wargelius et al., 2016). The gene *dnd1*, represented by gene family 13,707, is single copy across *Oncorhynchus* but is double copy in *S. salar* (Figure 5d). The high sequence divergence in one of the duplicates in *S. salar* is characteristic of rapid sequence evolution following duplication of *dnd1* which may suggest some functional divergence between the two paralogs. This may explain why targeting the one gene copy with lower sequence divergence is sufficient to block germ cell migration in these fish. As the field of genome editing in salmonids continues to grow, we hope that this homology guide will become a useful tool for planning CRISPR targeting strategies.

## 3 CONCLUSION

The relatively recent SS4R event makes salmonids an ideal system for studying the genomic consequences of WGD events. The SS4R has produced pronounced genetic variation, some of which has adaptive significance, which needs to be taken into account for practical applications of salmonid management and conservation. From an evolutionary prospective, we demonstrate that gene function is a major driver of whether ohnologs will be retained or will revert to a singleton state, which either conserves or erodes chromosomal synteny, respectively. Additionally, we demonstrate that while many ohnologs have developed divergent expression patterns, ohnologs are not more likely to promote adaptation than either paralogs or singleton genes in this system. Worth noting it that this may reflect differences in time scales as the divergence of these populations is much more recent than the SS4R and thus ohnologs may have already specialized prior to population divergence. From a practical application perspective, we provide an easy‐to‐use homology guide that provides the ohnolog/paralog/singleton status of each gene, gene family name, gene copy number, and NCBI gene ID across *Oncorhynchus* and *S. salar*. As salmonid biology moves into the functional genomics age this study should provide a basis for how the SS4R has shaped the evolutionary history of these six species and should influence the use of genomic‐based conservation strategies moving forward.

## 4 METHODS

### 4.1 Homology and ohnolog identification

The gene repertoire of each species was identified through downloading the predicted proteome and genome annotation file from NCBI for *O. tshawytscha* (Otsh_v2.0) (Christensen et al., 2018; Narum et al., 2018), *O. mykiss* (USDA_OmykA_1.1) (Gao et al., 2021), *O. nerka* (O. ner_1.0) (Christensen et al., 2020), *O. keta* (Oket_V1) (Rondeau et al., 2021), *O. kisutch* (Okis_V2) (Rondeau et al., 2022), *S. salar* (Ssal_v3.1), and *E. lucius* (fEsoLuc1.pri) (Ishiguro et al., 2003) in October 2021. *O. gorbuscha* (OgorEven_v1.0) (Christensen et al., 2021) was downloaded in February 2022. Gene isoforms were removed based on transcriptional start site and only the longest isoform was retained. After filtering out isoforms, we used Orthofinder2 (Emms & Kelly, 2015, 2019) to assign gene families and construct gene trees. Orthofinder2 has been shown to have the highest recall rate and among the highest levels of precision of any orthology assignment software and is particularly well suited for identifying non‐one‐to‐one homologous relationships and orthologs with high sequence divergence. The species tree was constructed using the program species tree from all genes (STAG) (Emms & Kelly, 2018) as implemented within Orthofinder2. Gene families were classified based upon gene counts and comparison to *E. lucius* (Northern Pike). Gene families were classified as ohnologs if the family was exactly twice as large in the focal species as in *E. lucius* and the two copies were on corresponding homoeologous chromosomes. A gene family was classified as a paralog if the family was exactly twice as large in the focal species as in *E. lucius* and the two copies were not on corresponding homoeologous chromosomes, which includes genes assigned to unplaced scaffolds. Of note, the delineation between ohnologs and paralogs were only made for gene families with two copies in the focal species and one copy in *E. lucius*. For larger gene families which were exactly twice as large in the focal species, these were called ohnologs. We adopted this strategy as identifying the correct correspondence between genes and homoeologous chromosomes scales exponentially with gene family size. Additionally, the majority of gene families which were twice as large in the focal species were on homoeologous chromosomes in the two copy gene families. While we acknowledge that this strategy would lead to false positives in larger gene families if the proportion of duplicate multi‐copy gene families is similar to single‐copy families, this would lead to 139 falsely identified ohnologs in *O. mykiss* and 337 in *O. tshawytscha* amounting to approximately 0.5% of *O. mykiss* genes and 1% of genes in *O. tshawytscha* which should have a negligible effect on our overall findings. Gene families which are in the process of resolving were larger in the focal species than *E. lucius* but less than twice as large. Gene families which have resolved to a singleton state were exactly the same size in the focal species and in *E. lucius*. While we use the term singleton to keep terminology consistent (Gundappa et al., 2022; Lien et al., 2016), this category includes some multi‐copy gene families. Expanding gene families were more than twice as large in the focal species compared with *E. lucius*. Contracting gene families were smaller in the focal species than they were in *E. lucius*.

### 4.2 GO enrichments

As our enrichment analysis were focused on identifying the term which were overrepresented among the gene families which were either universally retained as ohnologs or had reverted to a singleton state in *Oncorhynchus*, we decided to use the outgroup of *S. salar* as our enrichment background. We feel that *S. salar* is an ideal background as this species also experienced the SS4R WGD and has a high‐quality genome assembly. *S. salar* peptide sequences were annotated using the online portal of EggNOG mapper (Huerta‐Cepas et al., 2017, 2019) using taxa auto‐detection. *S. salar* annotations were used to assign gene names as well as GO terms to each gene family. GO enrichments were carried out for universally retained ohnologs across *Oncorhynchus* and universally singleton genes across *Oncorhynchus* separately. Enrichments were performed using the R script gene ontology with Mann–Whitney *U*‐test (GO_MWU) (Wright et al., 2017) using Fisher's exact test with *S. salar* as the enrichment background. This was accomplished by testing for terms overrepresented in the *S. salar* homologs of the *Oncorhynchus* genes which were universally retained ohnolog or singleton gene families compared with non‐ohnologs or non‐singletons respectively, for a total of two enrichment tests. For the ohnolog enrichments, genes which were universally retained as ohnologs were given a score of 1 while all other genes were given a score of 0. For the singleton enrichments, universally singleton genes were given a score of 1 while all other genes were given a score of 0. This strategy tests for functional enrichments among the ohnologs/singletons compared with genes which are not ohnologs/singletons, respectively, which serve as the genetic background. As pointed out by the reviewers, GO enrichments are sensitive to choice of enrichment background and we believe that the strategy employed by GO_MWU represents best practices in the field as it allows for the generation of a customized genetic background which can be tailored for the comparison being made. Additionally, this method minimizes phylogenetic biases which are common when using distantly related model species as a background (Wright et al., 2017). Identical parameters were used for each category (largest 0.25, smallest 50, clusterCutHeight = 0.25) and were kept consistent for both cellular compartment (CC) and molecular function (MF) enrichments. Terms were considered significant at a false discovery rate of 0.05 and a select subset was visualized for clarity purposes.

### 4.3 Synteny

Syntenic analysis was performed for three of the six species (*O. mykiss*, *O. tshawytscha*, and *O. nerka*) which includes one representative from each phylogenetic division within *Oncorhynchus*. Each species genome was initially separated by chromosome/linkage group and used as a query against the Atlantic salmon genome using LastZ (Harris, 2007) with parameters –chain –gapped –inner = 1000. LastZ alignments were then chained together with axtchain with parameters ‐minScore = 3000 ‐linearGap = medium, and then sorted using chainsort. Alignment chains were filtered with chainPreNet and then chained together with chainNet. Alignment nets were then made using netSyntenic. Nets were then converted into axt then maf format using netToAxt and axtToMaf, respectively. Finally, syntenic blocks were called using Maf2synteny with a minimum block size of 100 kb (Kolmogorov et al., 2018). Syntenic blocks were then combined if consecutive blocks were in the same strand orientation and the gap between two consecutive blocks was <1 Mb. This process was then repeated for each chromosome. Genes were considered to be in syntenic regions if the start site of a gene fell within syntenic blocks on the chromosome/linkage group it was on based on the annotation file. The relationship between ohnologs/singleton genes and syntenic regions was quantified using Fischer's exact test. All statistical analysis were carried in the R programming environment.

### 4.4 Local adaptation

The propensity of ohnologs to be associated with local adaptation was tested by reexamining RNA‐Seq data from the Redband trout system (Chen et al., 2018). Raw reads were downloaded from the NCBI short read archive SRP109007 and F1 hybrids were excluded from further analysis. Adaptors and low‐quality reads were removed with TrimGalore (Krueger, 2021). These filtered reads were then quantified using Salmon (Patro et al., 2017) with an index size of 31, decoys were constructed using genome sequences, and individual quantification files were combined using TXimport (Soneson et al., 2015). The read count table was then vst normalized in Deseq2 (Love et al., 2014) by population after removing transcripts with less than an average of 10 counts. Our generated count matrix and the phylogenetic tree produced from these populations based on whole‐genome resequencing data (Chen & Narum, 2021) were used in the Expression and Analysis of Variance model (Gillard et al., 2021; Rohlfs & Nielsen, 2015). This framework models expression level of a gene as a trait that evolves across a phylogeny using an Ornstein–Uhlenbeck process to determine a trait optima. As pointed out by the reviewers, the original implementation of the EVE model normalized read data with log‐transformed transcript‐per‐million values (Gillard et al., 2021), while our method relied on vst normalization of raw counts. This method has been shown to successfully normalize RNA‐Seq data for use in the EVE model (MacKnight et al., 2022), and we ensured that our residuals were normally distributed before proceeding. The variance from the trait optima both between and within populations is then compared with a beta‐shared test and significant variation is determined by a likelihood ratio test using chi‐squared distribution with one degree of freedom. After removing genes which did not converge on an optima, we compared the −log10 normalized beta statistic which we refer to as expression divergence (Dimos et al., 2022) with a one‐way ANOVA followed by Tukey's HSD multiple comparison correction.

## AUTHOR CONTRIBUTIONS

**Bradford Dimos:** Conceptualization (lead); formal analysis (lead); visualization (lead); writing – original draft (lead). **Michael Phelps:** Conceptualization (supporting); writing – review and editing (equal).

## Supporting information


**TABLE S1** Full results from GO enrichment analysis. Listed are all significant terms (FDR < 0.05) broken down by GO classification and if the enrichment was generated from universally retained ohnologs or universally singleton genes.

## Data Availability

All data and code need to replicate the results of this study are available on github: https://github.com/phelpslab. As well as on Figshare (https://figshare.com/articles/dataset/Data_for_A_Homology_Guide_for_Pacific_Salmon_Genus_Oncorhynchus_resolves_patterns_of_Duplicate_Retention_Rediploidization_and_Local_Adaptation_Following_the_Salmonid_Specific_Whole_Genome_Duplication_Event_/21290823). The homology guide can be found on both the Phelps' lab github: (https://github.com/phelpslab/Oncorhynchus_orthology) and website (https://mphelpslab.org/resources).
